# Supplementary material for: What is the best proxy for political knowledge in surveys?
Source: PLoS One. 2022 Aug 22;17(8):e0272530. doi: 10.1371/journal.pone.0272530 (PMC9394832; doi:10.1371/journal.pone.0272530)
Supplement: S1 File — (DOCX) [file pone.0272530.s001.docx]

**Tables S1a-f: Predictors of political knowledge and its proxies in the 2020 data using z-scores (Figure 1)**

*Table S1a: Political knowledge*

|  | Coef. | Robust SE | t | P>t | 95% conf. interval | |
| --- | --- | --- | --- | --- | --- | --- |
| Gender (male=1) | .5208294 | .0643464 | 8.09 | 0.000 | .3945719 | .647087 |
| Education | .8321386 | .0919748 | 9.05 | 0.000 | .6516699 | 1.012607 |
| Age | .9807674 | .1879586 | 5.22 | 0.000 | .6119639 | 1.349571 |
| Constant | -1.271486 | .1279464 | -9.94 | 0.000 | -1.522536 | -1.020435 |

Observations = 1,089
F(3, 1085) = 51.49
Prob > F = 0.0000
R-squared = 0.1478

*Table S1b: Self-assessment*

|  | Coef. | Robust SE | t | P>t | 95% conf. interval | |
| --- | --- | --- | --- | --- | --- | --- |
| Gender | .5936756 | .0856746 | 6.93 | 0.000 | .425569 | .7617823 |
| Education | .6884005 | .0796679 | 8.64 | 0.000 | .53208 | .8447211 |
| Age | .0977803 | .2242221 | 0.44 | 0.663 | -.3421778 | .5377384 |
| Constant | -.7627024 | .1400162 | -5.45 | 0.000 | -1.037435 | -.4879693 |

Observations = 1,089
F(3, 1085) = 55.90
Prob > F = 0.0000
R-squared = 0.0900

*Table S1c: Political interest*

|  | Coef. | Robust SE | t | P>t | 95% conf. interval | |
| --- | --- | --- | --- | --- | --- | --- |
| Gender | .328177 | .0665514 | 4.93 | 0.000 | .1975929 | .4587611 |
| Education | .7543874 | .097841 | 7.71 | 0.000 | .5624084 | .9463664 |
| Age | .3662338 | .1868935 | 1.96 | 0.050 | -.0004797 | .7329474 |
| Constant | -.8006675 | .1273282 | -6.29 | 0.000 | -1.050505 | -.5508301 |

Observations = 1,089
F(3, 1085) = 28.02
Prob > F = 0.0000
R-squared = 0.0739

*Table S1d: Internal political efficacy*

|  | Coef. | Robust SE | t | P>t | 95% conf. interval | |
| --- | --- | --- | --- | --- | --- | --- |
| Gender | .3247548 | .0661777 | 4.91 | 0.000 | .1949035 | .454606 |
| Education | .7390871 | .0943841 | 7.83 | 0.000 | .5538905 | .9242837 |
| Age | -.1729828 | .1826051 | -0.95 | 0.344 | -.5312831 | .1853174 |
| Constant | -.5198134 | .1277946 | -4.07 | 0.000 | -.7705668 | -.2690601 |

Observations = 1,086
F(3, 1082) = 30.33
Prob > F = 0.0000
R-squared = 0.0704

*Table S1e: Party placement*

|  | Coef. | Robust SE | t | P>t | 95% conf. interval | |
| --- | --- | --- | --- | --- | --- | --- |
| Gender | .4107481 | .0798617 | 5.14 | 0.000 | .254043 | .5674532 |
| Education | .7540913 | .1214907 | 6.21 | 0.000 | .5157015 | .9924811 |
| Age | .140489 | .2663219 | 0.53 | 0.598 | -.3820896 | .6630677 |
| Constant | -.7334011 | .2211495 | -3.32 | 0.001 | -1.167342 | -.29946 |

Observations = 1,089
F(3, 1085) = 31.43
Prob > F = 0.0000
R-squared = 0.0771

*Table S1f: Political participation*

|  | Coef. | Robust SE | t | P>t | [95% Conf. Interval] | |
| --- | --- | --- | --- | --- | --- | --- |
| Gender | -.0721019 | .0639566 | -1.13 | 0.260 | -.1975968 | .0533931 |
| Education | .9188101 | .0953363 | 9.64 | 0.000 | .7317423 | 1.105878 |
| Age | -1.18693 | .1845645 | -6.43 | 0.000 | -1.54908 | -.8247789 |
| Constant | .1028368 | .1284664 | 0.80 | 0.424 | -.1492387 | .3549122 |

Observations = 1,071
F(3, 1067) = 58.18
Prob > F = 0.0000
R-squared = 0.1347

**Tables S2a-e: Proxies as predictors of political knowledge in the 2020 data using z-scores (Figure 2)**

*Table S2a: Self-assessment*

|  | Coef. | Robust SE | t | P>t | 95% conf. interval | |
| --- | --- | --- | --- | --- | --- | --- |
| Self-assessment | .0412926 | .020637 | 2.00 | 0.046 | .0007997 | .0817856 |
| Gender | .0780859 | .015659 | 4.99 | 0.000 | .0473606 | .1088112 |
| Education | .1355005 | .0231118 | 5.86 | 0.000 | .0901516 | .1808495 |
| Age | .1891678 | .0366034 | 5.17 | 0.000 | .1173462 | .2609893 |
| Constant | .3885343 | .0312062 | 12.45 | 0.000 | .3273029 | .4497656 |

Observations = 1,089
F(4, 1084) = 42.36
Prob > F = 0.0000
R-squared = 0.2010

*Table S2b: Political interest*

|  | Coef. | Robust SE | t | P>t | 95% conf. interval | |
| --- | --- | --- | --- | --- | --- | --- |
| Political interest | .0805704 | .0061802 | 13.04 | 0.000 | .068444 | .0926969 |
| Gender | .076159 | .0115298 | 6.61 | 0.000 | .0535357 | .0987822 |
| Education | .1031451 | .0174029 | 5.93 | 0.000 | .068998 | .1372922 |
| Age | .1636978 | .0337998 | 4.84 | 0.000 | .0973773 | .2300182 |
| Constant | .4215504 | .0245871 | 17.15 | 0.000 | .3733067 | .4697942 |

Observations = 1,089
F(4, 1084) = 104.22
Prob > F = 0.0000
R-squared = 0.3010

*Table S2c: Internal political efficacy*

|  | Coef. | Robust SE | t | P>t | 95% conf. interval | |
| --- | --- | --- | --- | --- | --- | --- |
| Internal pol efficacy | .0473984 | .0064096 | 7.39 | 0.000 | .0348217 | .0599751 |
| Gender | .0888219 | .0124855 | 7.11 | 0.000 | .0643234 | .1133204 |
| Education | .1252307 | .0186852 | 6.70 | 0.000 | .0885673 | .1618941 |
| Age | .1991332 | .0357723 | 5.57 | 0.000 | .1289422 | .2693243 |
| Constant | .3844051 | .0247826 | 15.51 | 0.000 | .3357777 | .4330325 |

Observations = 1,086
F(4, 1081) = 63.93
Prob > F = 0.0000
R-squared = 0.2006

*Table S2d: Party placement*

|  | Coef. | Robust SE | t | P>t | 95% conf. interval | |
| --- | --- | --- | --- | --- | --- | --- |
| Party placement | .0381706 | .0072742 | 5.25 | 0.000 | .0238971 | .052444 |
| Gender | .0872468 | .0126282 | 6.91 | 0.000 | .0624676 | .1120259 |
| Education | .1268284 | .0184787 | 6.86 | 0.000 | .0905694 | .1630874 |
| Age | .1655227 | .0363419 | 4.55 | 0.000 | .0942124 | .2368331 |
| Constant | .4044878 | .0254349 | 15.90 | 0.000 | .3545792 | .4543963 |

Observations = 1,089
F(4, 1084) = 66.97
Prob > F = 0.0000
R-squared = 0.2174

*Table S2e: Participation*

|  | Coef. | Robust SE | t | P>t | 95% conf. interval | |
| --- | --- | --- | --- | --- | --- | --- |
| Participation | .0494595 | .0065047 | 7.60 | 0.000 | .0366959 | .062223 |
| Gender | .107912 | .0123588 | 8.73 | 0.000 | .0836617 | .1321622 |
| Education | .1201744 | .0186713 | 6.44 | 0.000 | .0835377 | .1568111 |
| Age | .2553955 | .0366312 | 6.97 | 0.000 | .183518 | .327273 |
| Constant | .3496735 | .0253095 | 13.82 | 0.000 | .3000114 | .3993357 |

Observations = 1,071
F(4, 1066) = 55.35
Prob > F = 0.0000
R-squared = 0.2010

**Tables 3a-o: Interactions between the proxies and sociodemographic controls (Figures 3-7)**

*Table 3a: Age and self-assessment*

|  | Coef. | SE | t | P>t | 95% conf. interval | |
| --- | --- | --- | --- | --- | --- | --- |
| Self-assessment | 1.11718 | .2551802 | 4.38 | 0.000 | .616479 | 1.61788 |
| Age group (ref. 18-30) |  |  |  |  |  |  |
| 31-45 | -1.120202 | .2995683 | -3.74 | 0.000 | -1.707999 | -.5324058 |
| 46-60 | -.4538216 | .3226163 | -1.41 | 0.160 | -1.086842 | .1791984 |
| 61- | -.0135737 | .2692368 | -0.05 | 0.960 | -.5418553 | .5147079 |
| Age group x self-assessment |  |  |  |  |  |  |
| 31-45 | 2.777829 | .4812263 | 5.77 | 0.000 | 1.833594 | 3.722065 |
| 46-60 | 1.959229 | .5390763 | 3.63 | 0.000 | .9014836 | 3.016975 |
| 61- | 1.620348 | .4287981 | 3.78 | 0.000 | .7789836 | 2.461712 |
| Constant | 4.346762 | .1804397 | 24.09 | 0.000 | 3.992714 | 4.700811 |

Observations = 1,097
F(7, 1089) = 36.22
Prob > F = 0.0000
Adj R-squared = 0.1836

*Table 3b: Age and political interest*

|  | Coef. | SE | t | P>t | 95% conf. interval | |
| --- | --- | --- | --- | --- | --- | --- |
| Political interest | 3.576865 | .4315134 | 8.29 | 0.000 | 2.730173 | 4.423556 |
| Age group (ref. 18-30) | |  |  |  |  |  |
| 31-45 | .6109685 | .3802526 | 1.61 | 0.108 | -.1351422 | 1.357079 |
| 46-60 | .698852 | .3960597 | 1.76 | 0.078 | -.0782746 | 1.475979 |
| 61- | 1.055412 | .3436591 | 3.07 | 0.002 | .3811035 | 1.729721 |
| Age group x political interest |  |  |  |  |  |  |
| 31-45 | -.5853529 | .5839424 | -1.00 | 0.316 | -1.731132 | .5604266 |
| 46-60 | -.4752125 | .6048007 | -0.79 | 0.432 | -1.661919 | .7114939 |
| 61- | -.6760247 | .5201296 | -1.30 | 0.194 | -1.696594 | .3445448 |
| Constant | 2.856042 | .276978 | 10.31 | 0.000 | 2.312571 | 3.399513 |

Observations = 1,097
F(7, 1089) = 45.70
Prob > F = 0.0000
Adj R-squared = 0.2221

*Table 3c: Age and internal political efficacy*

|  | Coef. | SE | t | P>t | 95% conf. interval | |
| --- | --- | --- | --- | --- | --- | --- |
| IPE | 2.680038 | .4407883 | 6.08 | 0.000 | 1.815145 | 3.544932 |
| Age group (ref. 18-30) | |  |  |  |  |  |
| 31-45 | .3727621 | .2896018 | 1.29 | 0.198 | -.1954803 | .9410045 |
| 46-60 | .8734231 | .2888443 | 3.02 | 0.003 | .306667 | 1.440179 |
| 61- | 1.368306 | .2603469 | 5.26 | 0.000 | .8574662 | 1.879146 |
| Age group x IPE |  |  |  |  |  |  |
| 31-45 | -.2665287 | .5705055 | -0.47 | 0.640 | -1.385947 | .8528892 |
| 46-60 | -.9806702 | .5642249 | -1.74 | 0.082 | -2.087765 | .1264242 |
| 61- | -1.297572 | .5235038 | -2.48 | 0.013 | -2.324766 | -.270379 |
| Constant | 3.862639 | .218352 | 17.69 | 0.000 | 3.434199 | 4.291078 |

Number of obs = 1,094
F(7, 1086) = 23.38
Prob > F = 0.0000
Adj R-squared = 0.1254

*Table 3d: Age and party placement*

|  | Coef. | SE | t | P>t | 95% conf. interval | |
| --- | --- | --- | --- | --- | --- | --- |
| Party placement | 5.949876 | 1.22739 | 4.85 | 0.000 | 3.541494 | 8.358258 |
| Age group (ref. 18-30) |  |  |  |  |  |  |
| 31-45 | .190355 | 1.772842 | 0.11 | 0.915 | -3.288313 | 3.669023 |
| 46-60 | -3.126321 | 2.003851 | -1.56 | 0.119 | -7.058275 | .8056327 |
| 61- | .7197284 | 1.479544 | 0.49 | 0.627 | -2.18343 | 3.622886 |
| Age group x party placement |  |  |  |  |  |  |
| 31-45 | .0868717 | 1.930831 | 0.04 | 0.964 | -3.701802 | 3.875545 |
| 46-60 | 3.861883 | 2.184385 | 1.77 | 0.077 | -.4243129 | 8.148079 |
| 61- | -.0000289 | 1.617002 | -0.00 | 1.000 | -3.172908 | 3.17285 |
| Constant | -.3536421 | 1.121866 | -0.32 | 0.753 | -2.554965 | 1.847681 |

Observations = 1,071
F(7, 1063) = 18.21
Prob > F = 0.0000
Adj R-squared = 0.1012

*Table 3e: Age and participation*

|  | Coef. | SE | t | P>t | 95% conf. interval | |
| --- | --- | --- | --- | --- | --- | --- |
| Participation | 3.623817 | .6714366 | 5.40 | 0.000 | 2.306337 | 4.941298 |
| Age group (ref. 18-30) | |  |  |  |  |  |
| 31-45 | .8164957 | .6061042 | 1.35 | 0.178 | -.3727906 | 2.005782 |
| 46-60 | 1.311385 | .5808714 | 2.26 | 0.024 | .1716094 | 2.45116 |
| 61- | 1.963976 | .4873658 | 4.03 | 0.000 | 1.007676 | 2.920276 |
| Age group x participation |  |  |  |  |  |  |
| 31-45 | -.9287644 | .9188836 | -1.01 | 0.312 | -2.731781 | .874252 |
| 46-60 | -1.334799 | .8867772 | -1.51 | 0.133 | -3.074817 | .4052185 |
| 61- | -1.558388 | .7736362 | -2.01 | 0.044 | -3.076402 | -.040373 |
| Constant | 2.731631 | .4339229 | 6.30 | 0.000 | 1.880195 | 3.583066 |

Observations = 1,079
F(7, 1071) = 17.82
Prob > F = 0.0000
Adj R-squared = 0.0985

*Table 3f: Gender and self-assessment*

|  | Coef. | SE | t | P>t | 95% conf. interval | |
| --- | --- | --- | --- | --- | --- | --- |
| Self-assessment | 2.562514 | .3326855 | 7.70 | 0.000 | 1.909739 | 3.215288 |
| Gender (male) | .865965 | .2222582 | 3.90 | 0.000 | .429864 | 1.302066 |
| Gender (male) x self-assessment | -.7348424 | .3957012 | -1.86 | 0.064 | -1.511262 | .0415775 |
| Constant | 3.898446 | .1676349 | 23.26 | 0.000 | 3.569523 | 4.227368 |

Observations = 1,097
F(3, 1093) = 65.22
Prob > F = 0.0000
Adj R-squared = 0.1495

*Table S3g: Gender and political interest*

|  | Coef. | SE | t | P>t | 95% conf. interval | |
| --- | --- | --- | --- | --- | --- | --- |
| Political interest | 2.290022 | .2640335 | 8.67 | 0.000 | 1.771952 | 2.808092 |
| Gender (male) | -.2867497 | .2471008 | -1.16 | 0.246 | -.7715952 | .1980958 |
| Gender (male) x political interest | 1.386475 | .3670279 | 3.78 | 0.000 | .6663163 | 2.106634 |
| Constant | 3.72322 | .1689219 | 22.04 | 0.000 | 3.391772 | 4.054668 |

Observations = 1,097
F(3, 1093) = 118.19
Prob > F = 0.0000
Adj R-squared = 0.2429

*Table S3h: Gender and internal political efficacy*

|  | Coef. | SE | t | P>t | 95% conf. interval | |
| --- | --- | --- | --- | --- | --- | --- |
| IPE | 1.499918 | .2475927 | 6.06 | 0.000 | 1.014105 | 1.98573 |
| Gender (male) | .4946295 | .1779474 | 2.78 | 0.006 | .1454713 | .8437878 |
| Gender (male) x IPE | .4826851 | .3460411 | 1.39 | 0.163 | -.1962969 | 1.161667 |
| Constant | 4.474499 | .1207862 | 37.04 | 0.000 | 4.2375 | 4.711499 |

Observations = 1,094
F(3, 1090) = 56.40
Prob > F = 0.0000
Adj R-squared = 0.1320

*Table S3i: Gender and party placement*

|  | Coef. | SE | t | P>t | 95% conf. interval | |
| --- | --- | --- | --- | --- | --- | --- |
| Party placement | 4.309154 | .7372428 | 5.84 | 0.000 | 2.862544 | 5.755765 |
| Gender (male) | -5.924289 | 1.456565 | -4.07 | 0.000 | -8.782347 | -3.066231 |
| Gender (male) x party placement | 7.14314 | 1.578219 | 4.53 | 0.000 | 4.046374 | 10.2399 |
| Constant | 1.225491 | .6693652 | 1.83 | 0.067 | -.0879308 | 2.538912 |

Observations = 1,071
F(3, 1067) = 54.56
Prob > F = 0.0000
Adj R-squared = 0.1306

*Table S3j: Gender and participation*

|  | Coef. | SE | t | P>t | 95% conf. interval | |
| --- | --- | --- | --- | --- | --- | --- |
| Participation | 1.772317 | .3672802 | 4.83 | 0.000 | 1.051649 | 2.492984 |
| Gender (male) | .3728312 | .3211688 | 1.16 | 0.246 | -.2573576 | 1.00302 |
| Gender (male) x participation | .7916012 | .5058942 | 1.56 | 0.118 | -.2010509 | 1.784253 |
| Constant | 3.999363 | .2378438 | 16.82 | 0.000 | 3.532672 | 4.466054 |

Observations = 1,079
F(3, 1075) = 45.55
Prob > F = 0.0000
Adj R-squared = 0.1103

*Table S3k: Education and self-assessment*

|  | Coef. | SE | t | P>t | 95% conf. interval | |
| --- | --- | --- | --- | --- | --- | --- |
| Self-assessment | 3.44956 | .6751725 | 5.11 | 0.000 | 2.12476 | 4.77436 |
| Education (ref. comprehensive) | |  |  |  |  |  |
| *Vocational* | -.3319417 | .3933481 | -0.84 | 0.399 | -1.103756 | .4398721 |
| *High school* | 1.358965 | .3847445 | 3.53 | 0.000 | .6040328 | 2.113897 |
| *Polytechnic* | .2416975 | .3966479 | 0.61 | 0.542 | -.5365911 | 1.019986 |
| *University* | .7004368 | .4291309 | 1.63 | 0.103 | -.1415888 | 1.542462 |
| Vocational x self-assessment | -.1348766 | .810967 | -0.17 | 0.868 | -1.726128 | 1.456374 |
| High school x self-assessment | -2.820695 | .725091 | -3.89 | 0.000 | -4.243443 | -1.397947 |
| Polytechnic x self-assessment | -.5044139 | .7889232 | -0.64 | 0.523 | -2.052412 | 1.043584 |
| University x self-assessment | -.5443717 | .80029 | -0.68 | 0.497 | -2.114673 | 1.025929 |
| Constant | 3.68598 | .3214733 | 11.47 | 0.000 | 3.055197 | 4.316764 |

Observations = 1,089
F(9, 1079) = 31.51
Prob > F = 0.0000
Adj R-squared = 0.2015

*Table S3l: Education and political interest*

|  | Coef. | SE | t | P>t | 95% conf. interval | |
| --- | --- | --- | --- | --- | --- | --- |
| Political interest | 3.066647 | .4702633 | 6.52 | 0.000 | 2.143913 | 3.989381 |
| Education (ref. comprehensive) | |  |  |  |  |  |
| *Vocational* | -.2678235 | .3656877 | -0.73 | 0.464 | -.985363 | .449716 |
| *High school* | -.5445159 | .5064487 | -1.08 | 0.283 | -1.538252 | .4492201 |
| *Polytechnic* | .0436371 | .3872759 | 0.11 | 0.910 | -.7162621 | .8035363 |
| *University* | 1.3618 | .4283359 | 3.18 | 0.002 | .5213346 | 2.202266 |
| Vocational x political interest | .0102291 | .5935114 | 0.02 | 0.986 | -1.154338 | 1.174797 |
| High school x political interest | .7698992 | .7612581 | 1.01 | 0.312 | -.7238148 | 2.263613 |
| Polytechnic x political interest | .0388783 | .6012559 | 0.06 | 0.948 | -1.140885 | 1.218642 |
| University x political interest | -1.164071 | .6276198 | -1.85 | 0.064 | -2.395565 | .0674224 |
| Constant | 3.474798 | .294318 | 11.81 | 0.000 | 2.897298 | 4.052299 |

Observations = 1,089
F(9, 1079) = 38.88
Prob > F = 0.0000
Adj R-squared = 0.2386

*Table S3m: Education and internal political efficacy*

|  | Coef. | SE | t | P>t | 95% conf. interval | |
| --- | --- | --- | --- | --- | --- | --- |
| IPE | 1.047473 | .5432536 | 1.93 | 0.054 | -.0184831 | 2.11343 |
| Education (ref. comprehensive) | |  |  |  |  |  |
| *Vocational* | -.5030568 | .288992 | -1.74 | 0.082 | -1.070109 | .0639949 |
| *High school* | -.5340022 | .390215 | -1.37 | 0.171 | -1.299671 | .2316664 |
| *Polytechnic* | .0757143 | .2859349 | 0.26 | 0.791 | -.485339 | .6367676 |
| *University* | .5911006 | .3284204 | 1.80 | 0.072 | -.0533165 | 1.235518 |
| Vocational x IPE | .6444433 | .6731314 | 0.96 | 0.339 | -.6763557 | 1.965242 |
| High school x IPE | 1.366077 | .7964323 | 1.72 | 0.087 | -.1966597 | 2.928813 |
| Polytechnic x IPE | .337215 | .6280699 | 0.54 | 0.591 | -.8951657 | 1.569596 |
| University x IPE | .3556134 | .6556914 | 0.54 | 0.588 | -.9309654 | 1.642192 |
| Constant | 4.824949 | .2370913 | 20.35 | 0.000 | 4.359735 | 5.290163 |

Observations = 1,086
F(9, 1076) = 17.99
Prob > F = 0.0000
Adj R-squared = 0.1235

*Table S3n: Education and party placement*

|  | Coef. | SE | t | P>t | 95% conf. interval | |
| --- | --- | --- | --- | --- | --- | --- |
| Party placement | 3.990748 | 1.285091 | 3.11 | 0.002 | 1.469118 | 6.512379 |
| Education (ref. comprehensive) | |  |  |  |  |  |
| *Vocational* | -1.12762 | 1.509334 | -0.75 | 0.455 | -4.089265 | 1.834024 |
| *High school* | -.763128 | 1.763177 | -0.43 | 0.665 | -4.222867 | 2.696611 |
| *Polytechnic* | -7.296863 | 2.475205 | -2.95 | 0.003 | -12.15376 | -2.439968 |
| *University* | -5.202608 | 2.394677 | -2.17 | 0.030 | -9.90149 | -.5037265 |
| Vocational x party placement | .8398189 | 1.68601 | 0.50 | 0.619 | -2.468503 | 4.148141 |
| High school x party placement | .9534172 | 1.949882 | 0.49 | 0.625 | -2.87268 | 4.779514 |
| Polytechnic x party placement | 8.039034 | 2.705539 | 2.97 | 0.003 | 2.730173 | 13.3479 |
| University x party placement | 6.365586 | 2.590211 | 2.46 | 0.014 | 1.283024 | 11.44815 |
| Constant | 1.719294 | 1.142 | 1.51 | 0.132 | -.5215608 | 3.960149 |

Observations = 1,063
F(9, 1053) = 18.77
Prob > F = 0.0000
Adj R-squared = 0.1309

*Table S3o: Education and participation*

|  | Coef. | SE | t | P>t | 95% conf. interval | |
| --- | --- | --- | --- | --- | --- | --- |
| Participation | 3.215144 | .7403619 | 4.34 | 0.000 | 1.762404 | 4.667884 |
| Education (ref. comprehensive) | |  |  |  |  |  |
| *Vocational* | .2200634 | .463714 | 0.47 | 0.635 | -.6898374 | 1.129964 |
| *High school* | .0664645 | .706125 | 0.09 | 0.925 | -1.319096 | 1.452025 |
| *Polytechnic* | 1.146979 | .4977879 | 2.30 | 0.021 | .1702182 | 2.123739 |
| *University* | 2.048697 | .5606127 | 3.65 | 0.000 | .9486611 | 3.148732 |
| Vocational x participation | -1.554928 | .8969875 | -1.73 | 0.083 | -3.314999 | .2051434 |
| High school x participation | -.7351705 | 1.171671 | -0.63 | 0.530 | -3.034225 | 1.563884 |
| Polytechnic x participation | -2.307258 | .9088983 | -2.54 | 0.011 | -4.0907 | -.5238156 |
| University x participation | -2.669895 | .9566984 | -2.79 | 0.005 | -4.547131 | -.7926593 |
| Constant | 3.777516 | .3621616 | 10.43 | 0.000 | 3.066882 | 4.488151 |

Observations = 1,071
F(9, 1061) = 13.97
Prob > F = 0.0000
Adj R-squared = 0.0984

Table S4: Factor score^1^ of PCA with all proxies as the predictor of political knowledge

|  | Coef. | SE | t | P>t | 95% conf. interval | |
| --- | --- | --- | --- | --- | --- | --- |
| Factor score | .0801825 | .0076128 | 10.53 | 0.000 | .0652442 | .0951208 |
| Gender (male = 1) | .0687076 | .0120261 | 5.71 | 0.000 | .0451094 | .0923058 |
| Education | .063963 | .0192175 | 3.33 | 0.001 | .0262533 | .1016726 |
| Age | .1948574 | .0346664 | 5.62 | 0.000 | .1268332 | .2628815 |
| Constant | .4384795 | .0265519 | 16.51 | 0.000 | .3863779 | .490581 |

^1^ Factor score for single factor derived from PCA including all the potential proxy measures. The coefficient is the z-standardized factor score, to ensure comparability with the other analyses.

Observations = 1,043
F(4, 1038) = 72.71
Prob > F = 0.0000
Adj. R-squared = 0.2854
