# Supplementary material for: What is the best proxy for political knowledge in surveys?
Source: PLoS One. 2022 Aug 22;17(8):e0272530. doi: 10.1371/journal.pone.0272530 (PMC9394832; doi:10.1371/journal.pone.0272530)
Supplement: S1 Appendix — (DOCX) [file pone.0272530.s002.docx]

**Appendix**

**Data description**

*2008 data*The following data description is from the Finnish Social Science Data Archive, where the data is deposited and available. The data identification code is FSD2499:
Target population: Finnish citizens aged 18 or over living in Finland, excluding the Åland Islands
Data collector(s): Taloustutkimus
Mode of data collection: Face-to-face interview
Sampling procedure: Probability; Stratified Quota sampling based on age, gender and municipality of residence.

*2020 data*The data is deposited in the Finnish Social Science Data Archive, from where it will be accessible for research.

Target population: Finnish citizens aged 18 or over living in Finland, excluding the Åland Islands
Data collector(s): Taloustutkimus
Mode of data collection: Face-to-face interview
Sampling procedure: Probability; Stratified Quota sampling based on age, gender and municipality of residence.

**Table A1. Variables**

| **Variable** | **Coding** | **Descriptives** (before rescaling) |
| --- | --- | --- |
| Political knowledge: *1) Where are the Powers of the State vested in Finland according to the Constitution? 2) Who is currently the Prime Minister of Finland and what party does (s)he represent? 3) Out of the following, please select the parties that currently form the Government 4) How often, at minimum, must parliamentary elections be held in Finland? 5) Who currently acts as the Speaker of the Parliament and what party does (s)he represent? 6) The cooperation of which two parties has been termed ’red earth/clay’? 7) Finland follows the so-called principle of parliamentarism. Which of the following best describes this principle? 8) How much is Finland’s annual government spending? 9) Annual inflation is 3% and Virtanen’s income increases by 2%. How does this affect Virtanen’s purchasing power?* | Correct = 1 Incorrect = 0 ‘Don’t know’ = 0; Not offered, but recorded if respondent did not provide an answer. Combined into an additive scale, then rescaled into values between 0 and 1.  Items 1, 4, 6, 7, 8 and 9 are multiple choice with four alternatives.  Item 2: Open-ended. Name and party needed for correct answer. Item 3: All government parties, and no other parties, needed for correct answer.  Item 5: Open-ended. Name and party needed for correct answer. | Min-max: 0-9  Mean: 2008=5.2  2020=5.5 |
| Self-assessment: *In your own assessment, how familiar are you with politics and societal matters?* | 1=very familiar 2=familiar 3=pretty familiar 4=not very familiar 5=not at all familiar 6=cannot say  Scale converted and rescaled into values between 0 and 1. For both 2008 and 2020, 4 ‘cannot say’ responses are excluded. | Min-max: 1-5  Mean: 2008=3.2 2020=3.0 |
| Political interest: *How interested are you in politics?* | 1=very interested 2=somewhat interested 3=not very interested 4=not at all interested 5=cannot say  Scale converted and rescaled into values between 0 and 1. | Min-max: 1-4 Mean: 2008=2.4 2020=2.1 |
| Internal political efficacy: *Sometimes politics seems so complicated that I can’t really understand what’s going on* | 1=completely disagree 2=partly disagree 3=partly agree 4 =completely agree 5=cannot say  Scale converted and rescaled into values between 0 and 1. | Min-max: 1-4 Mean: 2.3 |
| Party placement: *In politics, people often talk about left and right. On a scale where 0 means left and 10 means right, where would you place the following parties?* | Placement of all parliamentary parties: Social Democratic Party, National Coalition, Finns Party, Centre Party, Green League, Left Alliance, Swedish People’s Party and Christian Democrats. Grand total of the absolute differences between respondent and expert placement weighted by number of parties evaluated. Missing values assigned the arithmetic sample mean. Scale reversed so that higher value indicates shorter distance from expert evaluations in the 2019 Chapel Hill Survey. | Min-max: 0-1  Mean: .91 |
| Participation: 1) *Nowadays many people do not vote for some reason in elections. Did you vote in the latest parliamentary election?* 2) *The following is a list of different forms of societal participation. Which of the following have you done in the past four years or if you felt it was important, what might you do?* | 1) voted=1 did not vote=0 I was under aged=0 cannot say=0  2) For each form of participation: have done=1 might do=0.5 neither=0. Contacting a decision-maker Signing a citizen initiative Taking part in the activities of a political party Buying products on ethical basis Participating in peaceful demonstrations  All items combined into an additive scale, then rescaled into values between 0 and 1. | Min-max: 0-6  Mean: 3.6 |
| Age: *What year were you born?* | 2008 – [year] 2020 – [year]  Divided by 100. | Min-max:  2008: 18-91 2020: 18-90  Mean: 2008: 49 2020: 48 |
| Gender: *Are you female/male/do not want to say?* | 0=female 1=male  No missing values. | 2008: Female=51.6 % Male=48.4 % 2020: Female=51.2 % Male=48.8 % |
| Education: *What is the highest educational degree you have completed?* | 1=Primary education / lower secondary education  2=Vocational upper secondary education / vocational college  3=High school (general upper secondary education)  4=Polytechnic (upper vocational degree, university of applied sciences / polytechnic degree)  5=University degree (bachelor’s, master’s, licentiate or doctoral degree) | 2008:  Primary education=34.8 %  Vocational=24.8 %  High school=6.1 %  Polytechnic=22.6 %  University=11.7 %  2020:  Primary education=7.2 %  Vocational=17.9 %  High school=23.3 %  Polytechnic=36 %  University=15.7 % |

**Figure A1. Horn's parallel analysis plot**


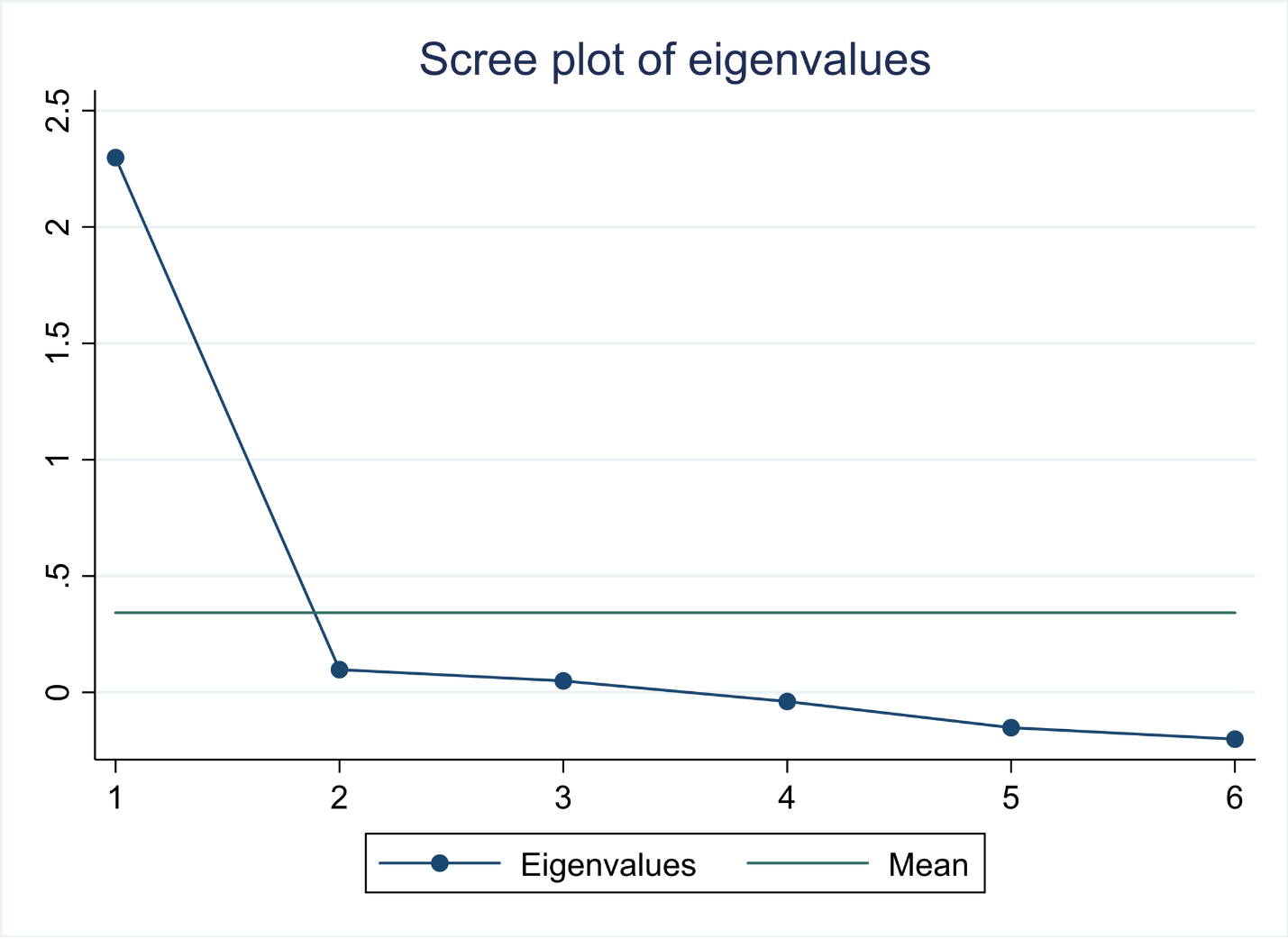


**Table A2. Correlation analysis with the 2008 data (Spearman rank order correlation, 95 % CIs, n=1,016)**

|  | Knowledge | Self-assessment | Political interest |
| --- | --- | --- | --- |
| Knowledge | - | .450 [.400 .498] | .408 [.355 .458] |
| Self-assessment | .450 [.400 .498] | - | .541 [.496 .583] |

Table A3. All proxies in the same model with the 2020 data (linear regression, n=1,097)

|  | Coef. | Std. Err. | T | P>t | 95% conf. interval |
| --- | --- | --- | --- | --- | --- |
| Self-assessment | .012951 | .0055298 | 2.34 | 0.019 | .0021001 .0238019 |
| Political interest | .0581189 | .0074202 | 7.83 | 0.000 | .0435584 .0726793 |
| IPE | .0161977 | .0061213 | 2.65 | 0.008 | .0041861 .0282092 |
| Party placement | .0233752 | .0063171 | 3.70 | 0.000 | .0109794 .035771 |
| Participation | .0066271 | .0068833 | 0.96 | 0.336 | -.0068798 .0201339 |
| Gender | .0655183 | .0123162 | 5.32 | 0.000 | .0413506 .089686 |
| Education | .068552 | .0184451 | 3.72 | 0.000 | .0323578 .1047461 |
| Age | .1625753 | .0366385 | 4.44 | 0.000 | .0906811 .2344696 |
| Constant | .451361 | .0269429 | 16.75 | 0.000 | .3984919 .50423 |

Table A4. All proxies in the same model with the 2008 data (linear regression, n = 1,015)

|  | Coef. | Std. Err. | T | P>t | 95% conf. interval |
| --- | --- | --- | --- | --- | --- |
| Self-assessment | .4920581 | .067149 | 7.33 | 0.000 | .3602903 .6238258 |
| Political interest | .3716881 | .0627524 | 5.92 | 0.000 | .2485479 .4948282 |
| Gender | .5828681 | .1106458 | 5.27 | 0.000 | .3657459 .7999902 |
| Education | 1.414376 | .1576778 | 8.97 | 0.000 | 1.104962 1.72379 |
| Age | 2.488583 | .3240554 | 7.68 | 0.000 | 1.852683 3.124483 |
| Constant | 3.200506 | .2051835 | 15.60 | 0.000 | 2.797871 3.603141 |

**Figure A2. Gender, age and education as predictors of knowledge and its proxies in the 2008 data (n=1,019, 95 % CIs)**


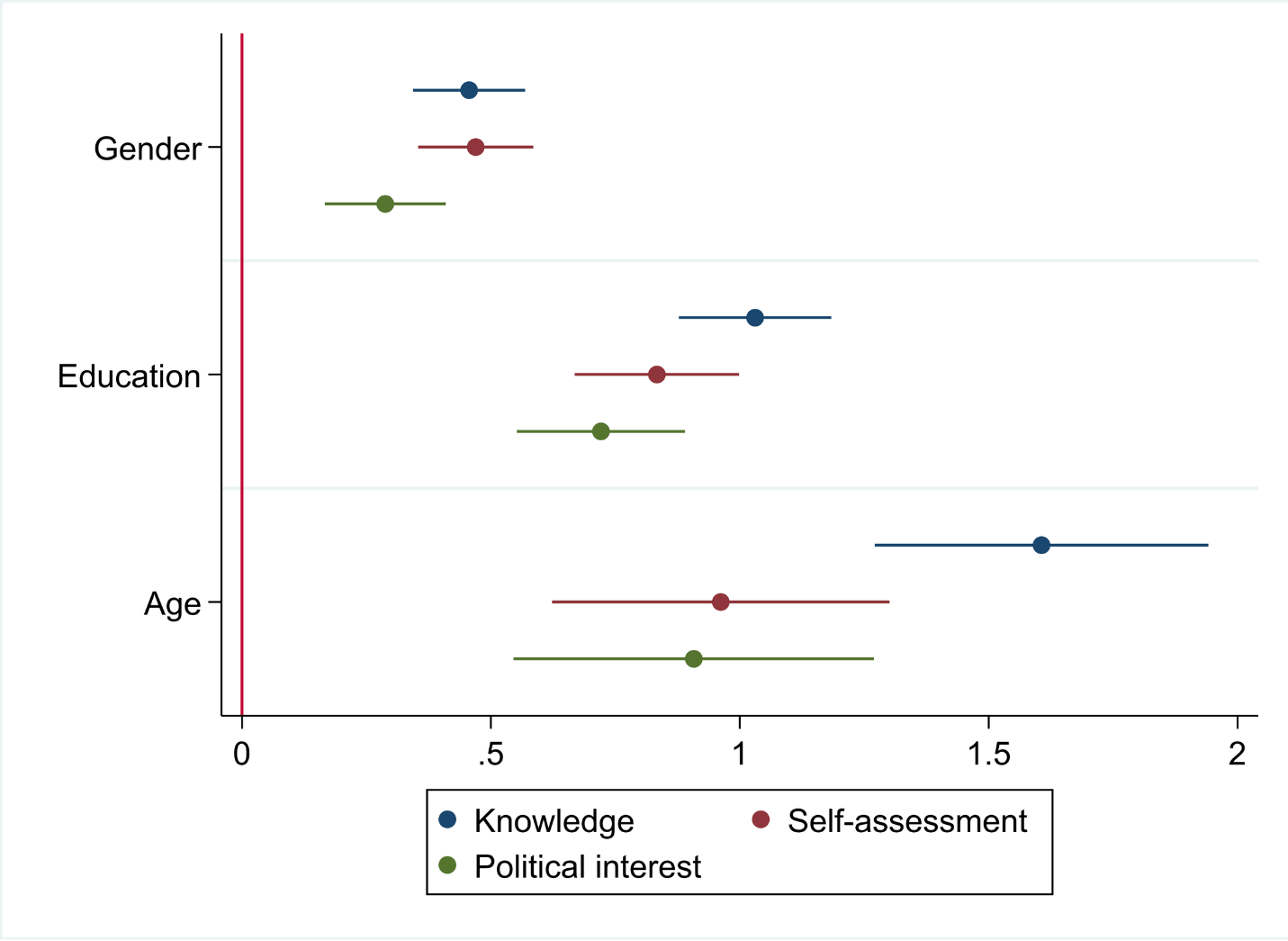


**Figure A3. Proxies as predictors of political knowledge in the 2008 data (n=1,019, 95 % CIs)**

**
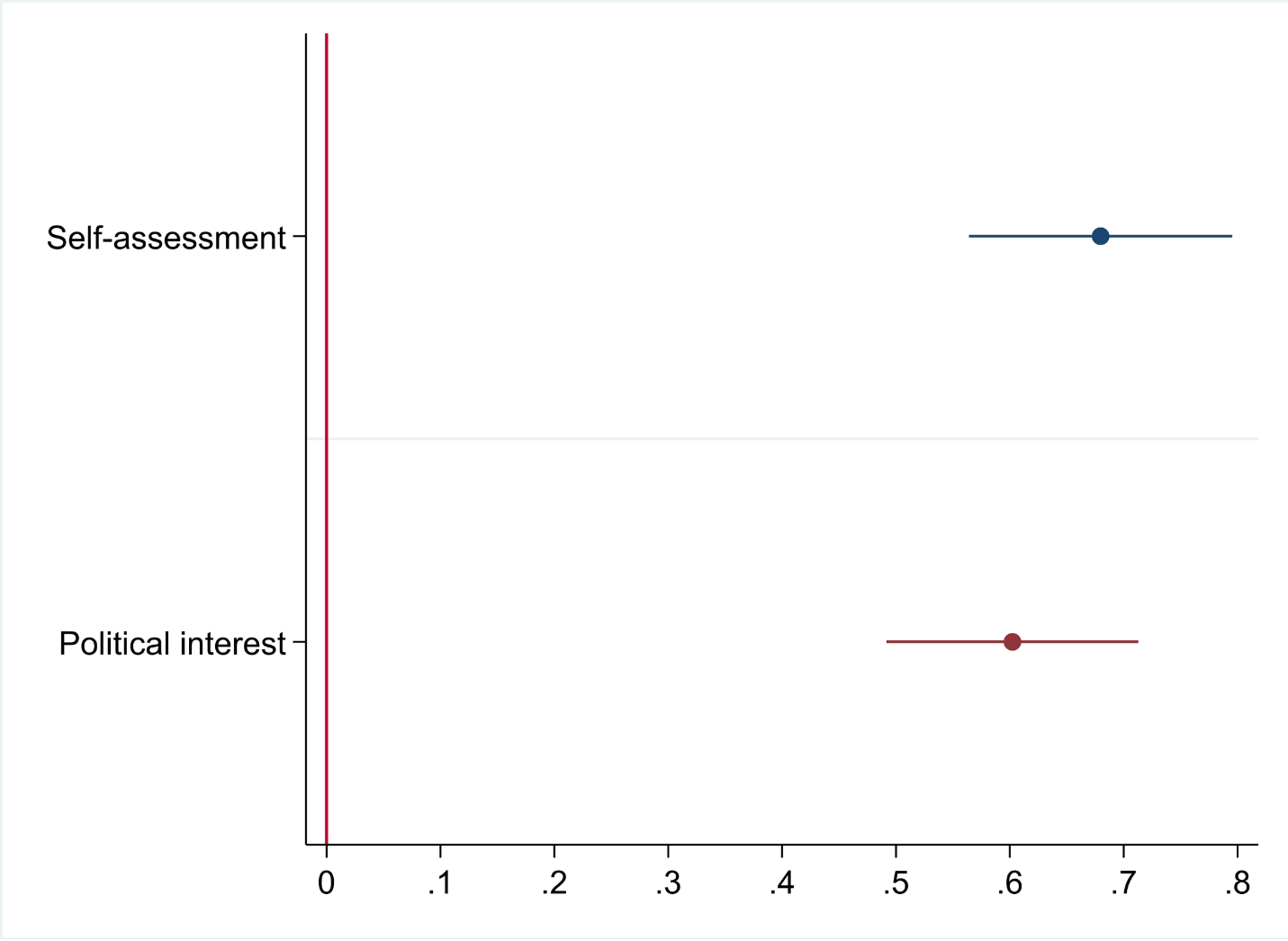
**
